# Retraction: Establishment and evaluation of the goose embryo epithelial (GEE) cell line as a new model for propagation of avian viruses

**DOI:** 10.1371/journal.pone.0305783

**Published:** 2024-06-13

**Authors:** The PLOS ONE Editors

After this article [1] was published, concerns were raised about results presented in Figs 1–3 and 5–7. Specifically:

- There appear to be similarities between Fig 1A in [1] and Fig 1A in [2] when rotated 180°.
- Fig 1C in [1] appears similar to parts of both panels in Fig 1A in [3], and to Fig 1C in [2].
- In Fig 2B, the DF-1 DAPI panel appears similar to the DEE DAPI panel when rotated 180°.
- There appear to be similarities between Fig 3D in [1] and Fig 6D in [3] when rotated 180°.
- The right two mice in the left panel of Fig 5C in [1] appear similar to the right two mice in the left panel of Fig 4B in [2] when stretched.
- The Fig 6A pcHA-GPV-VP3/DAPI panel appears similar to the Fig 6B pcHA/DAPI panel when rotated 180°.
- The three panels in Fig 7C in [1] appear similar to the three panels in Fig 5A in [2] when colors are adjusted.
- The three Mock panels across Figs 7A, B and C appear similar.

In response to queries about these experiments in Figure 7, the corresponding author stated that a single mock image was used for all three mock panels in Figs 7A, B and C since infection rate tests were conducted on the same cells. They provided individual-level data underlying the charts in Figs 4, 7 and 8, however, editorial assessment of these data raised further concerns regarding the validity, reliability, and integrity of these data.

In light of the extent of the concerns listed above that question the reliability and validity of the reported results and conclusions, the *PLOS ONE* Editors retract this article.

AS, BW, GQ, QX, BL and ZS either did not respond directly or could not be reached. WW did not agree with the retraction.

Owing to the concerns about similarities with previously published content [2], published in 2016 by Elsevier B.V., and [3], published in 2017 by Springer Science Business Media B.V., which are not offered under a CC BY license, Figs 1A, 1C, 3D, the left panel in Figure 5C, the Mock panels in Figs 7A-B and Figure 7C are excluded from this article’s [1] license. At the time of retraction, the article [1] was republished to note these exclusions in the legends of Figs 1, 3, 5 and 7 and the article’s copyright statement.
